# A case of severe malnutrition infant with neonatal onset intractable diarrhea

**DOI:** 10.1186/s12887-020-1999-0

**Published:** 2020-03-23

**Authors:** Youhong Fang, Youyou Luo, Jindan Yu, Jie Chen

**Affiliations:** Department of gastroenterology, The Children’s Hospital, Zhejiang University School of Medicine, National Clinical Research Center for Child Health, 3333 Bin Sheng Road, Hangzhou, 310052 Zhejiang Province China

**Keywords:** Chronic diarrhea, Growth failure, *EpCAM*, *TTC7A*

## Abstract

**Background:**

Congenital tufting enteropathy (CTE) is a rare disease that manifests as intractable diarrhea during the neonatal period which is associated with mutations of the epithelial cell adhesion molecule (*EpCAM*) gene.

**Case presentation:**

A male infant who presented with vomiting, diarrhea, abdominal distention, malnutrition and growth failure was admitted to our department when he was 2 months old. His parents were healthy and nonconsanguineous. Etiologic examinations of stool, inflammatory markers, blood gas and electrolytes levels, serum albumin level, serum immunoglobin levels were all normal. And there was no indication for metabolic diseases. Additionally, gastrointestinal contrast did not reveal abnormality of gastrointestinal. The patient was diagnosed with intestinal malabsorptive syndrome and severe malnutrition without definite cause. He was on supportive treatment and nutritional therapy for 13 months. However, he did not gain weight obviously. He was discharged at the age of 15 months and was fed with partial hydrolyzed formula and rice paste at home. Three months later he developed hypoglycemia and severe respiratory infection. Finally, he died due to sepsis and multiple organs failure. The next generation sequencing revealed one homozygous mutation of *EpCAM* gene and one complex heterozygous mutation of *TTC7A* gene. He was diagnosed CTE according to the genetic results and clinical manifestations.

**Conclusions:**

CTE is rarely reported in Asia. Patients present with congenital diarrhea, poor weight gain and growth failure are recommended to perform endoscopy examination with proper immunohistochemistry study as early as possible, and genetic testing is necessary when suspecting congenital diarrhea and enteropathy.

## Background

Congenital tufting enteropathy (CTE), also known as intestinal epithelial dysplasia or tufted bowel disease was first reported by Reifen et al. in 1994. In three cases of refractory diarrhea during neonatal period, the intestinal mucosal epithelial cells were abnormal. This was named tufted bowel disease [1]. The incidence of CTE is rare, with a prevalence of 1 in 50,000–100,000 in live births in Western Europe. The incidence rate is higher in areas with high proportion of close relatives and in the Arab region than in other regions [2, 3]. CTE is caused by epithelial cell adhesion molecule (*EpCAM)* mutation, and it is rare in Asia. Here we report an infant with neonatal onset chronic diarrhea and severe malnutrition caused by homozygous mutation of *EpCAM* gene.

## Case presentation

A male infant presented with vomiting, abdominal distension and watery diarrhea at the age of 2 weeks old. His vomiting was aggravated when he was 1 month old, and the episodes of vomiting were often occurred after feeding. At 2 months of age, he was admitted to our department. He was the first child of his mother, and he was born at term with a birth weight of 2.95 kg after an uneventful pregnancy. The parents were nonconsanguineous and healthy. There were no similar diseases occurred in his family history. When he was admitted, he had severe malnutrition, while his vital signs were stable. His weight was 3.42 kg, and the Z score of the weight for age (ZWFA) was below -3SD; his length was 55.5 cm, and the Z score of the height for age (ZHFA) was below -2SD. He had stable breathing, and lungs auscultation was normal, the rhythm and strength of the heart beat were normal. Neither abdominal tenderness nor hepatosplenomegaly was observed. No edema was observed. The complete blood count showed the following: a white blood cell count of 10.58 × 10^9^/L (65.7% lymphocytes), a hemoglobin level of 105 g/L, a platelet count of 628 × 10^9^ /L, a hypersensitive C-reactive protein level of 3 mg/L. The immunoglobulin (Ig) levels were within the normal range: Ig G 6 g/L, Ig A 0.36 g/L, Ig M 0.3 g/L, and Ig E < 17.9 IU/mL, and the serum albumin level and liver function were normal. The stool etiology examinations were all negative. Blood Analysis of blood tandem mass spectrometry did not indicate any metabolic disease. Gastrointestinal contrast revealed a horizontal transverse stomach and the ileum was segmental slightly extended. The patient was diagnosed with intestinal malabsorptive syndrome, severe malnutrition and growth failure according to the clinical and laboratory findings.

The patient was given partial parenteral nutrition and enteral nutrition with deep-hydrolysis formula during hospitalization. The daily amount of the stool was between 30 g per kilogram to 40 g per kilogram, and the largest amount of stool was 80 g per kilogram every day. The amount of stool decreased after hospitalization for 13 months, however there was only a minimal gain of weight from 3.42 kg to 4.88 kg, and the body height increased from 55.5 cm to 64.3 cm. The child was advised to perform an endoscopy procedure and genetic tests for suspecting congenital diarrhea and enteropathy. However, his parents refused to perform these examinations strongly. When he was discharged at 1-year-3-month old, the amount of formula he can take was about 700 ml per day. He was advised to continue parenteral nutrition treatment intermittently, but his parents did not take the doctor’s advice.

When the patient was 1 year and 6 months old, his diarrhea increased again. The episodes of stool were about five to six times a day. His mother added rice paste in his diet when he was 1 year and 8 months old. Half a month later, an edema was observed in the lower limbs and he had coughing as well. He was taken to the emergency room and the blood testing found hypoglycemia with blood glucose level of 1.2 mmol/L. Laboratory examinations showed the following: slightly decreased potassium ion level of 3.4 mmol/L, normal sodium ion level was normal, decreased calcium ion level of 0.56 mmol/L; a white blood cells count was 9.01 × 10^9^ /L (49.4% lymphocytes), a hemoglobin level was 122 g/L, platelets count was 202 × 10^9^ /L, normal hypersensitive C-reactive protein of 3 mg/L, normal serum albumin level of 34.4 g/L, slightly increased total bilirubin level of 31.0 μmol/L, increased alanine aminotransferase level of 119 U/L, an increased hypersensitive troponin T level of 0.165 ng/mL, an increased N-terminal B-type natriuretic peptide level more than 35,000 pg/mL. Chest X-ray showed pneumonia, multiple fractures of the ribs, narrowing of vertebral bodies and osteoporosis of various bones. An Echo ultrasound revealed decreased systolic function of left ventricular with EF level of 0.39. After admission, his general condition deteriorated quickly, and he developed sepsis and multiple organ failure. Finally, he had cardiac arrest and he needed repeated cardiopulmonary resuscitation. His parents gave up further treatment at last.

### Genetic analysis

Due to intractable diarrhea, growth failure and intestinal absorptive dysfunction, he was strongly suspected to have congenital diarrhea and entropy. He was advised to perform the genetic test again. Therefore, peripheral heparin anticoagulation blood was taken from him and his parents, and exon detection V2 which included over 4400 genes was performed at Beijing Mackinaw Medical Laboratory. The candidate genes of primary immunodeficiency diseases prone to refractory diarrhea including IL-2 receptor common gamma chain, *ZAP70*, *ORAI-1* and *ITK*, and those also associated with congenital diarrheas and enteropathies such as *DGAT1*, *SKIV2L*, *TTC7A*, *MYO5B*, *FOXP3*, *STAT3*, *IL10*, *IL10RA*, *IL10RB*, *NEMO*, and *STAT1* et al. were all analyzed. Genetic analysis revealed: two disease-related gene mutations in this patient, one homozygous mutation of *EpCAM* gene and the nucleotide number 491 + 1 in the coding region was changed from guanine to adenine, resulting in amino acid changes. One splicing mutation was derived from his mother, and his father was suspected to have a heterozygous deletion mutation of the gene. This homozygous gene mutation was associated with CTE and was functionally verified to be a causative gene [4]. The second mutation was a *TTC7A* gene complex heterozygous mutation, c.291_292insTACG, which was derived from his mother. He also had a mutation of c.335G > A, which was derived from his father. The compound heterozygous mutation was a suspected pathogenic mutation associated with multiple intestinal atresia (MIA) and chronic diarrhea. The patient was diagnosed with CTE (MIM 613217) in combination of clinical manifestations and genetic testing results.

## Discussion and conclusions

These patients of CTE mainly manifested as intractable diarrhea during the neonatal period, mainly for watery stool [2]. The pathogenesis is mainly related to the abnormal development or differentiation of intestine epithelial cells. The epithelial cells are destroyed and the cells are clustered in a specific cluster [5]. Often combined with other epithelial cell-related diseases or malformations. More than 60% of patients with punctate keratitis [2], combined with nostril atresia, esophageal atresia, no anus, etc. [2, 6].

The pathogenesis of CTE is associated with mutations in *EpCAM* [4]. Nearly 30 CTE patients with *EpCAM* mutations was reported up to 2008 [7]. The major mutations were located in the exon 3, 4, and 5 exon regions of the *EpCAM* gene, resulting in deletion of the extracellular and transmembrane regions of the EpCAM protein, thereby losing the function of anchoring to the serosa and failing to maintain normal intercellular barrier function [4, 7, 8]. The vast majority of reported *EpCAM* mutations caused CTE patients were from Europe, Northwest Africa, the Mediterranean and the Arab region, while two patients were from South Korea, and one patient was from China [7]. There are very few reports on CTE from Asia. The incidence of CTE in China and Asian population is unknown. CTE and other congenital diseases with diarrhea, bloating and growth failure are the main clinical manifestations. Several diseases are presented with similar clinical presentations, such as microvilli inclusion disease, congenital chlorine diarrhea. Patients with specific manifestations of CTE sometimes require repeated biopsy to detect, for patients with refractory diarrhea, highly suspected CTE patients can check mucosal biopsy with EpCAM immunohistochemical staining, and patients with immunohistochemistry considering CTE can further clarify the pathogenicity of EpCAM from the molecule level.

Currently there is no special treatment for CTE patients. Samuel Aquiline et al. reported that 13 patients with CTE were treated with long-term family parenteral nutrition [9]. Finally, 12 patients survived for 8 to 30 years under long-term parenteral nutrition therapy and the proportion of weaning off parenteral nutrition gradually increased with age. In the cases of inability to continue parenteral nutrition, end-stage liver disease, intestinal failure, and poor quality of life patients, bowel transplantation was attempted [2, 10].

**Fig. 1:**
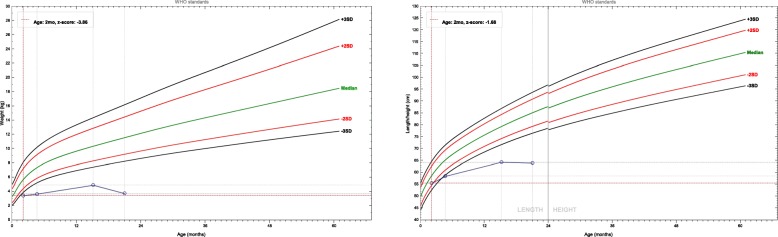
The growth chart of the patient revealed severe malnutrition and growth failure

The patient in this study developed diarrhea, bloating and vomiting 2 weeks after birth and there was an obvious weight loss and growth failure. He was diagnosed with intestinal malabsorptive syndrome without definite cause after the routine laboratory tests. When the patient was on continuous enteral nutrition and parenteral nutrition therapy, his weight did not increase significantly. After weaning off the parenteral nutrition, his condition become worse. The ZWFA and ZHFA of this child are shown in Fig. 1. At the second time the patient was admitted, he had growth failure, hypocalcemia, osteoporosis, multiple rib fractures, vertebral bones flattening and hypoglycemia. He eventually died due to respiratory infection, sepsis and multiple organs failure. The next generation sequencing result of the child demonstrated a homozygous mutation of *EpCAM* gene, and the pathogenicity was clear. The patients in this study harbored the compound heterozygous mutation of *TTC7A* gene as well, which is a suspected pathogenic mutation, and has not been reported previously. *TTC7A* mutation can be manifested as intestinal inflammation of very early inflammatory bowel disease [11]. However, this patient mainly presented with watery diarrhea and he had normal inflammatory markers. The phenotype of patients with *TTC7A* mutations included both MIA and combined T and/or B immunodeficiency (CID), both inflammatory bowel disease (IBD) and CID, isolated MIA, MIA, IBD, and CID complex, and isolated IBD [12]. This patient did not show MIA by gastrointestinal contras and the serum immunoglobin levels and the count of T and B cells were within normal range. The phonotype of this patient did not consistent with gene mutation. In this case, the efficacy of enteral and parenteral nutrition treatment was poor, and it is not clear whether it was related to the combined heterozygous mutation of *TTC7A*.

Overview the diagnosis process of this case, for patients with chronic diarrhea complicated with severe malnutrition and growth failure, endoscopic examination should be performed as soon as possible to obtain biopsy specimens of the intestinal mucosa which may provide information to the precise diagnosis. And the biopsy can be further examined by electron microscopy and immunohistochemistry conditionally, while next generation sequencing test can be suggested when congenital disease is suspected. Although refractory diarrhea caused by CTE is rare in Asia, we should aware this disease when patients preset with chronic diarrhea with early disease onset.

## Data Availability

There is no more case-specific data that could be shared.

## Abbreviations

CID: Combined T and/or B immunodeficiency
CTE: Congenital tufting enteropathy
EpCAM: Epithelial cell adhesion molecule
IBD: Inflammatory bowel disease
Ig: Immunoglobulin
MIA: Multiple intestinal atresia
ZHFA: Z score of the height for age
ZWFA: Z score of the weight for age

## References

[1] Reifen RM, Cutz E, Griffiths AM, Ngan BY, Sherman PM (1994). Tufting enteropathy: a newly recognized clinicopathological entity associated with refractory diarrhea in infants. J Pediatr Gastroenterol Nutr.

[2] Goulet O, Salomon J, Ruemmele F, de Serres NP, Brousse N (2007). Intestinal epithelial dysplasia (tufting enteropathy). Orphanet J Rare Dis.

[3] AlMahamed S, Hammo A (2017). New mutations of EpCAM gene for tufting enteropathy in Saudi Arabia. Saudi J Gastroenterol.

[4] Sivagnanam M, Mueller JL, Lee H, Chen Z, Nelson SF, Turner D, Zlotkin SH, Pencharz PB, Ngan BY, Libiger O (2008). Identification of EpCAM as the gene for congenital tufting enteropathy. Gastroenterology.

[5] Goulet OJ, Brousse N, Canioni D, Walker-Smith JA, Schmitz J, Phillips AD (1998). Syndrome of intractable diarrhoea with persistent villous atrophy in early childhood: a clinicopathological survey of 47 cases. J Pediatr Gastroenterol Nutr.

[6] Bird LM, Sivagnanam M, Taylor S, Newbury RO (2007). A new syndrome of tufting enteropathy and choanal atresia, with ophthalmologic, hematologic and hair abnormalities. Clin Dysmorphol.

[7] Tang W, Huang T, Xu Z, Huang Y (2018). Novel mutations in EPCAM cause congenital tufting Enteropathy. J Clin Gastroenterol.

[8] d'Apolito M, Pisanelli D, Faletra F, Giardino I, Gigante M, Pettoello-Mantovani M, Goulet O, Gasparini P, Campanozzi A (2016). Genetic analysis of Italian patients with congenital tufting enteropathy. World J Pediatr.

[9] Ashworth I, Wilson A, Aquilina S, Parascandalo R, Mercieca V, Gerada J, Macdonald S, Simchowitz V, Hill S (2018). Reversal of intestinal failure in children with tufting Enteropathy supported with parenteral nutrition at home. J Pediatr Gastroenterol Nutr.

[10] Paramesh AS, Fishbein T, Tschernia A, Leleiko N, Magid MS, Gondolesi GE, Kaufman SS (2003). Isolated small bowel transplantation for tufting enteropathy. J Pediatr Gastroenterol Nutr.

[11] Avitzur Y, Guo C, Mastropaolo LA, Bahrami E, Chen H, Zhao Z, Elkadri A, Dhillon S, Murchie R, Fattouh R (2014). Mutations in tetratricopeptide repeat domain 7A result in a severe form of very early onset inflammatory bowel disease. Gastroenterology.

[12] Lien R, Lin YF, Lai MW, Weng HY, Wu RC, Jaing TH, Huang JL, Tsai SF, Lee WI (2017). Novel mutations of the Tetratricopeptide repeat domain 7A gene and Phenotype/genotype comparison. Front Immunol.

